# When and how to update systematic reviews: consensus and checklist

**DOI:** 10.1136/bmj.i3507

**Published:** 2016-07-20

**Authors:** Paul Garner, Sally Hopewell, Jackie Chandler, Harriet MacLehose, Holger J Schünemann, Elie A Akl, Joseph Beyene, Stephanie Chang, Rachel Churchill, Karin Dearness, Gordon Guyatt, Carol Lefebvre, Beth Liles, Rachel Marshall, Laura Martínez García, Chris Mavergames, Mona Nasser, Amir Qaseem, Margaret Sampson, Karla Soares-Weiser, Yemisi Takwoingi, Lehana Thabane, Marialena Trivella, Peter Tugwell, Emma Welsh, Ed C Wilson, Panel for updating guidance for systematic reviews (PUGs)

**Affiliations:** 1 Cochrane Infectious Diseases Group, Department of Clinical Sciences, Liverpool School of Tropical Medicine, Liverpool L3 5QA, UK; 2 Oxford Clinical Trials Research Unit, University of Oxford, Oxford, UK; 3 Cochrane Editorial Unit, Cochrane Central Executive, London, UK; 4 Department of Clinical Epidemiology and Biostatistics and Department of Medicine, McMaster University, Hamilton, ON, Canada; 5 Cochrane GRADEing Methods Group, Ottawa, ON, Canada; 6 Department of Internal Medicine, American University of Beirut, Beirut, Lebanon; 7 Department of Mathematics and Statistics, McMaster University; 8 Evidence-based Practice Center Program, Agency for Healthcare and Research Quality, Rockville, MD, USA; 9 Centre for Reviews and Dissemination, University of York, York, UK; 10 Cochrane Upper Gastrointestinal and Pancreatic Diseases Group, Hamilton, ON, Canada; 11 Lefebvre Associates, Oxford, UK; 12 Kaiser Permanente National Guideline Program, Portland, OR, USA; 13 Iberoamerican Cochrane Centre, Barcelona, Spain; 14 Cochrane Informatics and Knowledge Management, Cochrane Central Executive, Freiburg, Germany; 15 Plymouth University Peninsula School of Dentistry, Plymouth, UK; 16 Department of Clinical Policy, American College of Physicians,****Philadelphia, PA, USA; 17 Guidelines International Network, Pitlochry, UK; 18 Children’s Hospital of Eastern Ontario, Ottawa, ON, Canada; 19 Institute of Applied Health Research, University of Birmingham, Birmingham, UK; 20 Biostatistics Unit, Centre for Evaluation, McMaster University, Hamilton, ON, Canada; 21 Centre for Statistics in Medicine, University of Oxford, Oxford, UK; 22 University of Ottawa, Ottawa, ON, Canada; 23 Cochrane Airways Group, Population Health Research Institute, St George’s, University of London, London, UK; 24 Cambridge Centre for Health Services Research, University of Cambridge, Cambridge, UK

## Abstract

Updating of systematic reviews is generally more efficient than starting all over again when new evidence emerges, but to date there has been no clear guidance on how to do this. This guidance helps authors of systematic reviews, commissioners, and editors decide when to update a systematic review, and then how to go about updating the review.

Systematic reviews synthesise relevant research around a particular question. Preparing a systematic review is time and resource consuming, and provides a snapshot of knowledge at the time of incorporation of data from studies identified during the latest search. Newly identified studies can change the conclusion of a review. If they have not been included, this threatens the validity of the review, and, at worst, means the review could mislead. For patients and other healthcare consumers, this means that care and policy development might not be fully informed by the latest research; furthermore, researchers could be misled and carry out research in areas where no further research is actually needed.^1^ Thus, there are clear benefits to updating reviews, rather than duplicating the entire process as new evidence emerges or new methods develop. Indeed, there is probably added value to updating a review, because this will include taking into account comments and criticisms, and adoption of new methods in an iterative process.^2^ ^3^ ^4^ ^5^ ^6^

Cochrane has over 20 years of experience with preparing and updating systematic reviews, with the publication of over 6000 systematic reviews. However, Cochrane’s principle of keeping all reviews up to date has not been possible, and the organisation has had to adapt: from updating when new evidence becomes available,^7^ to updating every two years,^8^ to updating based on need and priority.^9^ This experience has shown that it is not possible, sensible, or feasible to continually update all reviews all the time. Other groups, including guideline developers and journal editors, adopt updating principles (as applied, for example, by the *Systematic Reviews* journal; https://systematicreviewsjournal.biomedcentral.com/). 

The panel for updating guidance for systematic reviews (PUGs) group met to draw together experiences and identify a common approach. The PUGs guidance can help individuals or academic teams working outside of a commissioning agency or Cochrane, who are considering writing a systematic review for a journal or to prepare for a research project. The guidance could also help these groups decide whether their effort is worthwhile.

**Summary points**

Updating systematic reviews is, in general, more efficient than starting afresh when new evidence emerges. The panel for updating guidance for systematic reviews (PUGs; comprising review authors, editors, statisticians, information specialists, related methodologists, and guideline developers) met to develop guidance for people considering updating systematic reviews. The panel proposed the following:

1. Decisions about whether and when to update a systematic review are judgments made for individual reviews at a particular time. These decisions can be made by agencies responsible for systematic review portfolios, journal editors with systematic review update services, or author teams considering embarking on an update of a review.
2. The decision needs to take into account whether the review addresses a current question, uses valid methods, and is well conducted; and whether there are new relevant methods, new studies, or new information on existing included studies. Given this information, the agency, editors, or authors need to judge whether the update will influence the review findings or credibility sufficiently to justify the effort in updating it.
3. Review authors and commissioners can use a decision framework and checklist to navigate and report these decisions with “update status” and rationale for this status. The panel noted that the incorporation of new synthesis methods (such as Grading of Recommendations Assessment, Development and Evaluation (GRADE)) is also often likely to improve the quality of the analysis and the clarity of the findings.
4. Given a decision to update, the process needs to start with an appraisal and revision of the background, question, inclusion criteria, and methods of the existing review.
5. Search strategies should be refined, taking into account changes in the question or inclusion criteria. An analysis of yield from the previous edition, in relation to databases searched, terms, and languages can make searches more specific and efficient.
6. In many instances, an update represents a new edition of the review, and authorship of the new version needs to follow criteria of the International Committee of Medical Journal Editors (ICMJE). New approaches to publishing licences could help new authors build on and re-use the previous edition while giving appropriate credit to the previous authors.

The panel also reflected on this guidance in the context of emerging technological advances in software, information retrieval, and electronic linkage and mining. With good synthesis and technology partnerships, these advances could revolutionise the efficiency of updating in the coming years.

## Panel selection and procedures

An international panel of authors, editors, clinicians, statisticians, information specialists, other methodologists, and guideline developers was invited to a two day workshop at McMaster University, Hamilton, Canada, on 26-27 June 2014, organised by Cochrane. The organising committee selected the panel (web appendix 1). The organising committee invited participants, put forward the agenda, collected background materials and literature, and drafted the structure of the report.

The purpose of the workshop was to develop a common approach to updating systematic reviews, drawing on existing strategies, research, and experience of people working in this area. The selection of participants aimed on broad representation of different groups involved in producing systematic reviews (including authors, editors, statisticians, information specialists, and other methodologists), and those using the reviews (guideline developers and clinicians). Participants within these groups were selected on their expertise and experience in updating, in previous work developing methods to assess reviews, and because some were recognised for developing approaches within organisations to manage updating strategically. We sought to identify general approaches in this area, and not be specific to Cochrane; although inevitably most of the panel were somehow engaged in Cochrane.

The workshop structure followed a series of short presentations addressing key questions on whether, when, and how to update systematic reviews. The proceedings included the management of authorship and editorial decisions, and innovative and technological approaches. A series of small group discussions followed each question, deliberating content, and forming recommendations, as well as recognising uncertainties. Large group, round table discussions deliberated further these small group developments. Recommendations were presented to an invited forum of individuals with varying levels of expertise in systematic reviews from McMaster University (of over 40 people), widely known for its contributions to the field of research evidence synthesis. Their comments helped inform the emerging guidance.

The organising committee became the writing committee after the meeting. They developed the guidance arising from the meeting, developed the checklist and diagrams, added examples, and finalised the manuscript. The guidance was circulated to the larger group three times, with the PUGs panel providing extensive feedback. This feedback was all considered and carefully addressed by the writing committee. The writing committee provided the panel with the option of expressing any additional comments from the general or specific guidance in the report, and the option for registering their own view that might differ to the guidance formed and their view would be recorded in an annex. In the event, consensus was reached, and the annex was not required.

## Definition of update

The PUGs panel defined an update of a systematic review as a new edition of a published systematic review with changes that can include new data, new methods, or new analyses to the previous edition. This expands on a previous definition of a systematic review update.^10^ An update asks a similar question with regard to the participants, intervention, comparisons, and outcomes (PICO) and has similar objectives; thus it has similar inclusion criteria. These inclusion criteria can be modified in the light of developments within the topic area with new interventions, new standards, and new approaches. Updates will include a new search for potentially relevant studies and incorporate any eligible studies or data; and adjust the findings and conclusions as appropriate. Box 1 provides some examples.

**Box 1: Examples of what factors might change in an updated systematic review**

- A systematic review of steroid treatment in tuberculosis meningitis used GRADE methods and split the composite outcome in the original review of death plus disability into its two components. This improved the clarity of the reviews findings in relation to the effects and the importance of the effects of steroids on death and on disability.^11^
- A systematic review of dihydroartemisinin-piperaquine (DHAP) for treating malaria was updated with much more detailed analysis of the adverse effect data from the existing trials as a result of questions raised by the European Medicines Agency. Because the original review included other comparisons, the update required extracting only the DHAP comparisons from the original review, and a modification of the title and the PICO.^12^
- A systematic review of atorvastatin was updated with simple uncontrolled studies.^13^ This update allowed comparisons with trials and strengthened the review findings.^14^

## Which systematic reviews should be updated and when?

Any group maintaining a portfolio of systematic reviews as part of their normative work, such as guidelines panels or Cochrane review groups, will need to prioritise which reviews to update. Box 2 presents the approaches used by the Agency for HealthCare Research and Quality (AHRQ) and Cochrane to prioritise which systematic reviews to update and when. Clearly, the responsibility for deciding which systematic reviews should be updated and when they will be updated will vary: it may be centrally organised and resourced, as with the AHRQ scientific resource centre (box 2). In Cochrane, the decision making process is decentralised to the Cochrane Review Group editorial team, with different approaches applied, often informally.

**Box 2: Examples of how different organisations decide on updating systematic reviews**

**Agency for Healthcare Research and Quality (US)**

The AHRQ uses a needs based approach; updating systematic reviews depends on an assessment of several criteria:

1. Stakeholder impact
  - Interest from stakeholder partners (such as consumers, funders, guideline developers, clinical societies, James Lind Alliance)
  - Use and uptake (for example, frequency of citations and downloads)
  - Citation in scientific literature including clinical practice guidelines
2. Currency and need for update
  - New research is available
  - Review conclusions are probably dated
3. Update decision
  - Based on the above criteria, the decision is made to either update, archive, or continue surveillance.

**Cochrane**

Of over 50 Cochrane editorial teams, most but not all have some systems for updating, although this process can be informal and loosely applied. Most editorial teams draw on some or all of the following criteria:

1. Strategic importance
  - Is the topic a priority area (for example, in current debates or considered by guidelines groups)?
  - Is there important new information available?
2. Practicalities in organising the update that many groups take into account
  - Size of the task (size and quality of the review, and how many new studies or analyses are needed)
  - Availability and willingness of the author team
3. Impact of update
  - New research impact on findings and credibility
  - Consider whether new methods will improve review quality
4. Update decision
  - Priority to update, postpone update, class review as no longer requiring an update

The PUGs panel recommended an individualised approach to updating, which used the procedures summarised in figure 1. The figure provides a status category, and some options for classifying reviews into each of these categories, and builds on a previous decision tool and earlier work developing an updating classification system.^15^ ^16^ We provide a narrative for each step.

**Figure f1:**
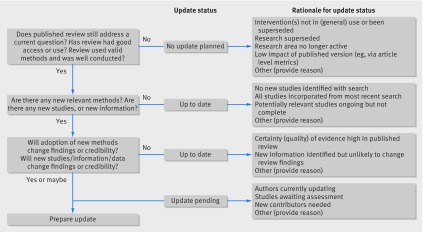
**Fig 1** Decision framework to assess systematic reviews for updating, with standard terms to report such decisions

## Step 1: assess currency

### Does the published review still address a current question?

An update is only worthwhile if the question is topical for decision making for practice, policy, or research priorities (fig 1). For agencies, people responsible for managing a portfolio of systematic reviews, there is a need to use both formal and informal horizon scanning. This type of scanning helps identify questions with currency, and can help identify those reviews that should be updated. The process could include monitoring policy debates around the review, media outlets, scientific (and professional) publications, and linking with guideline developers.

### Has the review had good access or use?

Metrics for citations, article access and downloads, and sharing via social or traditional media can be used as proxy or indicators for currency and relevance of the review. Reviews that are widely cited and used could be important to update should the need arise. Comparable reviews that are never cited or rarely downloaded, for example, could indicate that they are not addressing a question that is valued, and might not be worth updating.

In most cases, updated reviews are most useful to stakeholders when there is new information or methods that result in a change in findings. However, there are some circumstances in which an up to date search for information is important for retaining the credibility of the review, regardless of whether the main findings would change or not. For example, key stakeholders would dismiss a review if a study is carried out in a relevant geographical setting but is not included; if a large, high profile study that might not change the findings is not included; or if an up to date search is required for a guideline to achieve credibility. Box 3 provides such examples. If the review does not answer a current question, the intervention has been superseded, then a decision can be made not to update and no further intelligence gathering is required (fig 1).

**Box 3: Examples of a systematic review’s currency**

- The public is interested in vitamin C for preventing the common cold: the Cochrane review includes over 29 trials with either no or small effects, concluding good evidence of no important effects.^17^ Assessment: still a current question for the public.
- Low osmolarity oral rehydration salt (ORS) solution versus standard solution for acute diarrhoea in children: the 2001 Cochrane review^18^ led the World Health Organization to recommend ORS solution formula worldwide to follow the new ORS solution formula^19^ and this has now been accepted globally. Assessment: no longer a current question.
- Routine prophylactic antibiotics with caesarean section: the Cochrane review reports clear evidence of maternal benefit from placebo controlled trials but no information on the effects on the baby.^20^ Assessment: this is a current question.
- A systematic review published in the *Lancet* examined the effects of artemisinin based combination treatments compared with monotherapy for treating malaria and showed clear benefit.^21^ Assessment: this established the treatment globally and is no longer a current question and no update is required.
- A Cochrane review of amalgam restorations for dental caries^22^ is unlikely to be updated because the use of dental amalgam is declining, and the question is not seen as being important by many dental specialists. Assessment: no longer a current question.

### Did the review use valid methods and was it well conducted?

If the question is current and clearly defined, the systematic review needs to have used valid methods and be well conducted. If the review has vague inclusion criteria, poorly articulated outcomes, or inappropriate methods, then updating should not proceed. If the question is current, and the review has been cited or used, then it might be appropriate to simply start with a new protocol. The appraisal should take into account the methods in use when the review was done.

## Step 2: identify relevant new methods, studies, and other information

### Are there any new relevant methods?

If the question is current, but the review was done some years ago, the quality of the review might not meet current day standards. Methods have advanced quickly, and data extraction and understanding of the review process have become more sophisticated. For example:

- Methods for assessing risk of bias of randomised trials,^23^ diagnostic test accuracy (QUADAS-2),^24^ and observational studies (ROBINS-1).^25^
- Application of summary of findings, evidence profiles, and related GRADE methods has meant the characteristics of the intervention, characteristics of the participants, and risk of bias are more thoroughly and systematically documented.^26^ ^27^
- Integration of other study designs containing evidence, such economic evaluation and qualitative research.^28^

There are other incremental improvements in a wide range of statistical and methodological areas, for example, in describing and taking into account cluster randomised trials.^29^ AMSTAR can assess the overall quality of a systematic review,^30^ and the ROBIS tool can provide a more detailed assessment of the potential for bias.^31^

### Are there any new studies or other information?

If an authoring or commissioning team wants to ensure that a particular review is up to date, there is a need for routine surveillance for new studies that are potentially relevant to the review, by searching and trial register inspection at regular intervals. This process has several approaches, including:

- Formal surveillance searching^32^
- Updating the full search strategies in the original review and running the searches
- Tracking studies in clinical trial and other registers
- Using literature appraisal services^33^
- Using a defined abbreviated search strategy for the update^34^
- Checking studies included in related systematic reviews.^35^

How often this surveillance is done, and which approaches to use, depend on the circumstances and the topic. Some topics move quickly, and the definition of “regular intervals” will vary according to the field and according to the state of evidence in the field. For example, early in the life of a new intervention, there might be a plethora of studies, and surveillance would be needed more frequently.

## Step 3: assess the effect of updating the review

### Will the adoption of new methods change the findings or credibility?

Editors, referees, or experts in the topic area or methodologists can provide an informed view of whether a review can be substantially improved by application of current methodological expectations and new methods (fig 1). For example, a Cochrane review of iron supplementation in malaria concluded that there was “no significant difference between iron and placebo detected.”^36^ An update of the review included a GRADE assessment of the certainty of the evidence, and was able to conclude with a high degree of certainty that iron does not cause an excess of clinical malaria because the upper relative risk confidence intervals of harm was 1.0 with high certainty of evidence.^37^

### Will the new studies, information, or data change the findings or credibility?

The assessment of new data contained in new studies and how these data might change the review is often used to determine whether an update should go ahead, and the speed with which the update should be conducted. The appraisal of these new data can be carried out in different ways. Initially, methods focused on statistical approaches to predict an overturning of the current review findings in terms of the primary or desired outcome (table 1). Although this aspect is important, additional studies can add important information to a review, which is more than just changing the primary outcome to a more accurate and reliable estimate. Box 4 gives examples.

**Table 1 tbl1:** Formal prediction tools: how potentially relevant new studies can affect review conclusions

Method	Description of approach	How it could be used	Advantages	Limitations	Validation
GRADE approach^38^	Considers whether the evidence certainty might change in the update (for example, because of lack of high certainty evidence, or because new evidence contradicts existing high certainty evidence). High certainty of evidence for critical outcomes could lower the priority for updating. Uncertainty in the review findings increases the need to include new studies^38^	Provides a benchmark by outcome to assess whether a new trial will improve the certainty of the evidence	Pragmatic. Many reviews already include GRADE	Requires GRADE to have been used in existing review or to complete an assessment according to GRADE	GRADE summary of findings tables or evidence profiles widely validated. Use of GRADE approach to prioritising updates requires further validation
Ottawa method^39^-^41^	A simple PubMed search (using the three largest and three most recent trials from the original review) to identify new studies. If new studies are found, then the method uses quantitative signals (eg, change in significance, effect size) to assess the likelihood that the new studies will change the review conclusion, thus triggering an update	Practical routine surveillance tool	Easy to use	Will not detect all trials; judgment only based on changing conclusion	Approach validated for consistency of predicted and actual changes to conclusions; reasonable agreement with RAND method^39^ ^42^-^44^
RAND method^45^	An abbreviated search of five major journals to identify new studies, and a search of the US Food and Drug Administration website and external expert judgment to determine the currency of the report findings	Practical routine surveillance tool	Easy to use	Will not detect all trials; judgment only based on changing conclusion	Approach validated for consistency of predicted and actual changes to conclusions, and compares well with the Ottawa method^39^ ^43^ ^44^
Statistical prediction tool^15^	A multicomponent decision tool to assess whether there might be any new studies for the update. If new studies are identified, a statistical prediction tool estimates the probability that this will change the review conclusion	Ranks multiple systematic reviews in order of priority for updating	Uses quantitative approach	More complicated; requires commercial software	Approach validated internally^46^; requires further external validation
Value of information analysis^47^ ^48^	Builds on the statistical prediction tool approach^15^ comparing the expected health gain from new evidence with its expected cost. The gain is calculated in terms of a reduction in expected loss from reduced uncertainty and the cost is measured in days required to update the review.	Ranks selected systematic reviews in order of priority for updating	Uses quantitative approach	More complicated; requires some statistical knowledge	Approach validated internally; requires further external validation

**Box 4: Examples of new information other than new trials being important**

- The iconic Cochrane review of steroids in preterm labour was thought to provide evidence of benefit in infants, and this question no longer required new trials. However, a new large trial published in the* Lancet* in 2015 showed that in low and middle income countries, strategies to promote the uptake of neonatal steroids increased neonatal mortality and suspected maternal infection.^49^ This information needs to somehow be incorporated into the review to maintain its credibility.
- A Cochrane review of community deworming in developing countries indicates that in recent studies, there is little or no effect.^50^ The inclusion of a large trial of two million children confirmed that there was no effect on mortality. Although the incorporation of the trial in the review did not change the review’s conclusions, the trial’s absence would have affected the credibility of the review, so it was therefore updated.
- A new paper reporting long term follow-up data on anthracycline chemotherapy as part of cancer treatment was published. Although the effects from the outcomes remained essentially unchanged, apart from this longer follow-up, the paper also included information about the performance bias in the original trial, shifting the risk of bias for several outcomes from “unknown” to “high” in the Cochrane review.^51^

Reviews with a high level of certainty in the results (that is, when the GRADE assessment for the body of evidence is high) are less likely to change even with the addition of new studies, information, or data, by definition. GRADE can help guide priorities in whether to update, but it is still important to assess new studies that might meet the inclusion criteria. New studies can show unexpected effects (eg, attenuation of efficacy) or provide new information about the effects seen in different circumstances (eg, groups of patients or locations).

Other tools are specifically designed to help decision making in updating. For example, the Ottawa^39^ and RAND^45^ methods focus on identification of new evidence, the statistical predication tool^15^ calculates the probability of new evidence changing the review conclusion, and the value of information analysis approach^52^ calculates the expected health gain (table 1). As yet, there has been limited external validation of these tools to determine which approach would be most effective and when.

If potentially relevant studies are identified that have not previously been assessed for inclusion, authors or those managing the updating process need to assess whether including them might affect the conclusions of the review. They need to examine the weight and certainty of the new evidence to help determine whether an update is needed and how urgent that update is. The updating team can assess this informally by judging whether new studies or data are likely to substantively affect the review, for example, by altering the certainty in an existing comparison, or by generating new comparisons and analyses in the existing review.

New information can also include fresh follow-up data on existing included studies, or information on how the studies were carried out. These should be assessed in terms of whether they might change the review findings or improve its credibility (fig 1). Indeed, if any study has been retracted, it is important the authors assess the reasons for its retraction. In the case of data fabrication, the study needs to be removed from the analysis and this recorded. A decision needs to be made as to whether other studies by the same author should be removed from the review and other related reviews. An investigation should also be initiated following guidelines from the Committee on Publication Ethics (COPE). Additional published and unpublished data can become available from a wide range of sources—including study investigators, regulatory agencies and industry—and are important to consider.

## Preparing for an update

### Refresh background, objectives, inclusion criteria, and methods

Before including new studies in the review, authors need to revisit the background, objectives, inclusion criteria, and methods of the current review. In Cochrane, this is referred to as the protocol, and editors are part of this process. The update could range from simply endorsing the current question and inclusion criteria, through to full rewriting of the question, inclusion criteria and methods, and republishing the protocol. As a field progresses with larger and better quality trials rigorously testing the questions posed, it may be appropriate to exclude weaker study designs (such as quasi-randomised comparisons or very small trials) from the update (table 2). The PUGs panel recommended that a protocol refresh will require the authors to use the latest accepted methods of synthesis, even if this means repeating data extraction for all studies.

**Table 2 tbl2:** Refresh background, objectives, inclusion criteria, and methods

Protocol section	Appraisal points
Background and research question	• Review and update background section, including supporting references to take account of any changes that may have occurred. This should include updating any new information and current policy debates on the topic. • Assess whether the current review question remains relevant to patients and practice.
Inclusion criteria	• Consider whether the existing PICO(s) remain(s) current, in the light of new knowledge. • Identify any new understanding of definition of patient populations. • Identify new interventions, or those that have been withdrawn, that are no longer in use. • Identify any changes in usual care standards. • Check for standardised core outcomes sets, such as those developed in collaboration with the core outcome measures in effectiveness trials (COMET) initiative (www.comet-initiative.org) or by guideline groups since the original review. • Check for any relevant patient reported outcomes to include subsequent to the original review. • Consider any new studies with less risk of bias that might warrant a stricter study design inclusion criteria (where the older version, when there was a dearth of evidence, included observational or quasi-randomised comparisons).
Methods	• Appraise and update the methods pending relevant methodological advancements or developments. For example, if (1) there are new tools for assessing the risk of bias of individual studies or appraising the quality of a body of evidence (eg, GRADE); or (2) new and efficient search approaches are feasible, such as a targeted approach to searching, taking into account the quality of the original search, and ensuring that the search for the update is of high quality. • Update or include a summary of findings table, which is recommended for all systematic reviews, because it improves the clarity, understanding, and interpretation of the findings of a systematic review, and rapidly reduces the amount of time readers require to find key information.^53^-^55^

### New authors and authorship

Updated systematic reviews are new publications with new citations. An authorship team publishing an update in a scientific or medical journal is likely to manage the new edition of a review in the same way as with any other publication, and follow the ICMJE authorship criteria.^56^ If the previous author or author team steps down, then they should be acknowledged in the new version. However, some might perceive that their efforts in the first version warrant continued authorship, which may be valid. The management of authorship between versions can sometimes be complicated. At worst, it delays new authors completing an update and leads to long authorship lists of people from previous versions who probably do not meet ICMJE authorship criteria. One approach with updates including new authors is to have an opt-in policy for the existing authors: they can opt in to the new edition, provided that they make clear their contribution, and this is then agreed with the entire author team.

Although they are new publications, updates will generally include content from the published version. Changing licensing rights around systematic reviews to allow new authors of future updates to remix, tweak, or build on the contributions of the original authors of the published version (similar to the rights available via a Creative Commons licence; https://creativecommons.org) could be a more sustainable and simpler approach. This approach would allow systematic reviews to continue to evolve and build on the work of a range of authors over time, and for contributors to be given credit for contributions to this previous work.

### Efficient searching

In performing an update, a search based on the search conducted for the original review is required. The updated search strategy will need to take into account changes in the review question or inclusion criteria, for example, and might be further adjusted based on knowledge of running the original search strategy. The search strategy for an update need not replicate the original search strategy, but could be refined, for example, based on an analysis of the yield of the original search. These new search approaches are currently undergoing formal empirical evaluation, but they may well provide much more efficient search strategies in the future. Some examples of these possible new methods for review updates are described in web appendix 2.

In reporting the search process for the update, investigators must ensure transparency for any previous versions and the current update, and use an adapted flow diagram based on PRISMA reporting (preferred reporting items for systematic reviews and meta-analyses).^57^ The search processes and strategies for the update must be adequately reported such that they could be replicated.

### Peer review

Systematic reviews published for the first time in peer reviewed journals are by definition peer reviewed, but practice for updates remains variable, because an update might have few changes (such as an updated search but no new studies found and therefore included) or many changes (such as revise methods and inclusion of several new studies leading to revised conclusions). Therefore, and to use peer reviewers’ time most effectively, editors need to consider when to peer review an update and the type of peer reviewer most useful for a particular update (for example, topic specialist, methodologist). The decision to use peer review, and the number and expertise of the peer reviewers could depend on the nature of the update and the extent of any changes to the systematic review as part of an editor assessment. A change in the date of the search only (where no new studies were identified) would not require peer review (except, arguably, peer review of the search), but the addition of studies that lead to a change in conclusions or significant changes to the methods would require peer review. The nature of the peer review could be described within the published article.

### Reporting changes

Authors should provide a clear description of the changes in approach or methods between different editions of a review. Also, authors need to report the differences in findings between the original and updated edition to help users decide how to use the new edition. The approach or format used to present the differences in findings might vary with the target user group.^58^ Publishers need to ensure that all previous versions of the review remain publically accessible.

Updates can range from small adjustments to reviews being completely rewritten, and the PUGs panel spent some time debating whether the term “new edition” would be a better description than “update.” However, the word “update” is now in common parlance and changing the term, the panel judged, could cause confusion. However, the debate does illustrate that an update could represent a review that asks a similar question but has been completely revised.

## Technology and innovation

The updating of systematic review is generally done manually and is time consuming. There are opportunities to make better use of technology to streamline the updating process and improve efficiency (table 3). Some of these tools already exist and are in development or in early use, and some are commercially available or freely available. The AHRQ’s evidence based practice centre team has recently published tools for searching and screening, and will provide an assessment of the use, reliability, and availability of these tools.^63^

**Table 3 tbl3:** Technological innovations to improve the efficiency of updating systematic reviews

Innovation	Description	Application	Examples of software and projects,* and current status
Integrated software	Integration of applying inclusion criteria, review management systems, statistical packages, and GRADE	To facilitate greater efficiencies in review production, including their updates	Covidence (www.covidence.org): free/pay† EPPI reviewer (http://eppi.ioe.ac.uk/cms/Default.aspx): pay DistillerSR (https://distillercer.com/products/distillersr-systematic-review-software/): pay Cochrane Review Manager (http://tech.cochrane.org/revman): free/pay† GRADEpro GDT (http://gradepro.org/): free Rayyan (http://rayyan.qcri.org/): free
Systematic review data repositories	Repositories store information from review (eg, data abstraction forms and the evidence tables)	Improve updating efficiency for new or existing teams as the data abstraction forms, evidence tables, and populating data from the original review are available	Agency of Health Care Research and Quality systematic review data repository (http://srdr.ahrq.gov): operational GRADE database of evidence profiles and evidence to decision frameworks (http://dbep.gradepro.org/): operational
Semi-automation	Machine learning techniques to use alongside human efforts	Finding studies and extracting data could benefit from semi-automation creating time efficiencies^59^	RobotReviewer (http://vortext.systems/robotreviewer): free Cochrane project transform—identification of studies (http://community.cochrane.org/tools/project-coordination-and-support/transform)
Crowdsourcing	Use of volunteers to assist systematic review authors with discrete tasks	Individuals from the “crowd” assist with tasks (identifying and screening studies, translating articles, data extraction) to help in new review production and updates^60^-^62^	Cochrane Project Transform—crowdsourcing (link as above)
Publication linkage	Ability to link trial registration, trial publications, and reviews citing them will help transparency	This initiative could help identify studies for systematic reviews and could also show the relation between systematic review updates	A cross publisher initiative, CrossRef, is coordinating a threaded publications/linked clinical trial reports initiative to link a clinical trial report (with a trial registration number) report and derivative publications, including reviews (www.crossref.org): operational
Data linkage	Increase links between data, existing software, and reviews	To improve identification and reuse of data for review production and dissemination	Cochrane (http://linkeddata.cochrane.org/): proof of principle example at production phase, but mostly linkage projects at exploratory phase

*Further information can be located on the SR Toolbox site (http://systematicreviewtools.com/).

†Free to Cochrane contributors; other users pay.

Other developments, such as targeted updates that are performed rapidly and focus on updating only key components of a review, could provide different approaches to updating in the future and are being piloted and evaluated.^64^ With implementation of these various innovations, the longer term goal is for “living” systematic reviews, which identify and incorporate information rapidly as it evolves over time.^60^

## Concluding remarks

Updating systematic reviews, rather than addressing the same question with a fresh protocol, is generally more efficient and allows incremental improvement over time. Mechanical rules appear unworkable, but there is no clear unified approach on when to update, and how implement this. This PUGs panel of authors, editors, statisticians, information specialists, other methodologists, and guideline developers brought together current thinking and experience in this area to provide guidance.

Decisions about whether and when to update a systematic review are judgments made at a point in time. They depend on the currency of the question asked, the need for updating to maintain credibility, the availability of new evidence, and whether new research or new methods will affect the findings.

Whether the review uses current methodological standards is important in deciding if the update will influence the review findings, quality, reliability, or credibility sufficiently to justify the effort in updating it. Those updating systematic reviews to author clinical practice guidelines might consider the influence of new study results in potentially overturning the conclusions of an existing review. Yet, even in cases where new study findings do not change the primary outcome measure, new studies can carry important information about subgroup effects, duration of treatment effects, and other relevant clinical information, enhancing the currency and breadth of review results.

An update requires appraisal and revision of the background, question, inclusion criteria, and methods of the existing review and the existing certainty in the evidence. In particular, methods might need to be updated, and search strategies reconsidered. Authors of updates need to consider inputs to the current edition, and follow ICMJE criteria regarding authorship.^56^

The PUGs panel proposed a decision framework (fig 1), with terms and categories for reporting the decisions made for updating procedures for adoption by Cochrane and other stakeholders. This framework includes journals publishing systematic review updates and independent authors considering updates of existing published reviews. The panel developed a checklist to help judgements about when and how to update.

The current emphasis of authors, guideline developers, Cochrane, and consequently this guidance has been on effects reviews. The checklists and guidance here still applies to other types of systematic reviews, such as those on diagnostic test accuracy, and this guidance will need adapting. Accumulative experience and methods development in reviews other than those of effects are likely to help refine guidance in the future.

This guidance could help groups identify and prioritise reviews for updating and hence use their finite resources to greatest effect. Software innovation and new management systems are being developed and in early use to help streamline review updates in the coming years.

## Web Extra

Extra material supplied by the author

**Web appendix:** Supplementary material

## Notes

Provenance and peer review: Not commissioned; externally peer reviewed.
