# When and how to update systematic reviews: consensus and checklist

**DOI:** 10.1136/bmj.i4853

**Published:** 2016-09-06

On initial online publication of this Research Methods and Reporting article (*BMJ* 2016;354:i3507, doi:10.1136/bmj.i3507), Holger J Schünemann should have been at the end of the author list - this has since been corrected.

