# Supplementary material for: When and how to update systematic reviews: consensus and checklist
Source: BMJ. 2016 Jul 20;354:i3507. doi: 10.1136/bmj.i3507 (PMC4955793; doi:10.1136/bmj.i3507)
Supplement: Supplementary file 1 — Web appendix: Supplementary material [file garp030756.ww_default.pdf]

Garner P, Hopewell S, Chandler J, MacLehose H, Schünemann H, for the panel for updating guidance for systematic reviews (PUGs). When and how to update systematic reviews: consensus and checklist. British Medical Journal 2016: supplementary material.

APPENDIX 1. LIST OF WORKSHOP PARTICIPANTS

Name	Affiliation
Elie A. Akl	Professor, Department of Internal Medicine, American University of Beirut, Beirut (Lebanon); Cochrane GRADEing Methods Group (Canada) ea32@aub.edu.lb
Joseph Beyene	Associate Professor, Department of Mathematics and Statistics, McMaster University (Canada) beyene@mcmaster.ca
Jackie Chandler*	Methods Co-ordinator, Cochrane Editorial Unit, Cochrane Central Executive (UK)
Stephanie Chang	Director, Evidence-based Practice Center Program, Agency for Healthcare and Research Quality, Rockville (USA) Stephanie.Chang@ahrq.hhs.gov
Rachel Churchill	Professor, Centre for Reviews and Dissemination, University of York (UK); Cochrane Common Mental Disorders Group, York (UK) Rachel.Churchill@york.ac.uk
Karin Dearness	McMaster University (Canada); Managing Editor, Cochrane Upper Gastrointestinal and Pancreatic Diseases Group (Canada) dearnes@mcmaster.ca
Paul Garner*	Professor, Co-ordinating Editor Cochrane Infectious Diseases Group (UK); Evidence Synthesis for Global Health, Department of Clinical Sciences, Liverpool School of Tropical Medicine, Liverpool (UK)
Gordon Guyatt	Professor, Department of Clinical Epidemiology & Biostatistics, McMaster University (Canada) guyatt@mcmaster.ca
Sally Hopewell*	Associate Professor, Oxford Clinical Trials Research Unit, University of Oxford (UK); Cochrane Methodology Review Group (UK)
Carol Lefebvre	Information Consultant, Lefebvre Associates, Oxford (UK); Co-Convenor, Cochrane Information Retrieval Methods Group (UK) carol@lefebvreassociates.org
Beth Liles	Methodologist, Kaiser Permanente National Guideline Program, Portland (USA) Elizabeth.G.Liles@kp.org
Harriet MacLehose*	Senior Editor, Cochrane Editorial Unit, Cochrane Central Executive (UK)
Rachel Marshall	Editor, Cochrane Editorial Unit, Cochrane Central Executive (UK) rmarshall@cochrane.org
Laura Martínez García	Researcher, Iboamerican Cochrane Centre (Spain) laura.martinez.garcia@cochrane.es
Chris Mavergames	Head of Cochrane Informatics and Knowledge Management, Cochrane Central Executive (Germany) cmavergames@cochrane.org
David Moher	Senior Scientist, Clinical Epidemiology, Ottawa Hospital Research Institute (Canada); Associate Professor, Department of Epidemiology and Community Medicine, Faculty of Medicine, University of Ottawa. (Canada)
Mona Nasser	Clinical Lecturer in Evidence Based Dentistry, Plymouth University Peninsula School of Dentistry (UK); Co-Convenor, Cochrane Priority Setting Methods Group (UK) mona.nasser@plymouth.ac.uk
Amir Qaseem	Vice President, Department of Clinical Policy, American College of Physicians (USA); Chair of the Guidelines International Network (G-I-N) (UK) aqaseem@acponline.org
Margaret Sampson	Librarian, Children's Hospital of Eastern Ontario (Canada); Member, Cochrane Information Retrieval Methods Group (Canada) msampson@cheo.on.ca

Holger J. Schünemann*	Professor and Chair, Department of Clinical Epidemiology and Biostatistics, and Department of Medicine, McMaster University (Canada); Cochrane GRADEing Methods Group (Canada)
Karla Soares-Weiser	Deputy Editor in Chief, Cochrane Editorial Unit, Cochrane Central Executive (UK) ksoares-weiser@cochrane.org
Yemisi Takwoingi	Senior Research Fellow in Medical Statistics, Institute of Applied Health Research, University of Birmingham (UK); Co-Convenor, Cochrane Screening and Diagnostic Tests Methods Group (UK) y.takwoingi@bham.ac.uk
Lehana Thabane	Director and Professor, Biostatistics Unit, Centre for Evaluation, McMaster University (Canada); Professor, Department of Clinical Epidemiology & Biostatistics, McMaster University (Canada) thabanl@mcmaster.ca
Marialena Trivella	Statistician, Centre for Statistics in Medicine, University of Oxford (UK); Statistician and Editor, Cochrane Anaesthesia, Critical and Emergency Care Group (UK) marialena.trivella@csm.ox.ac.uk
Peter Tugwell	Professor of Medicine, and Epidemiology and Community Medicine, University of Ottawa (Canada); Co-ordinating Editor, Cochrane Musculoskeletal Group (Canada) maria.cannataro@uottawa.ca
Emma Welsh	Managing Editor, Cochrane Airways Group, Population Health Research Institute, St George's, University of London (UK) ewelsh@sgul.ac.uk
Ed C. Wilson	Co-Convenor, Cochrane Priority Setting Methods and Campbell & Cochrane Economic Methods Groups; Senior Research Associate in Health Economics, Cambridge Centre for Health Services Research, University of Cambridge (UK) Ed.Wilson@medschl.cam.ac.uk
*Member of organizing committee and writing group	

APPENDIX 2. SEARCH STRATEGIES FOR A SYSTEMATIC REVIEW UPDATE

Newly emerging research is helping refine searching for studies for systematic review updates. This is based on an analysis of original searches and the yield in relation to databases searched, terms, and languages to improve the specificity of searches and reduce the burden of author screening. Further research will help clarify the effectiveness and efficiency of these innovations.

Refining the original searches

Database selection and search strategy optimization

Database selection: While the original search may include multiple databases of many journals, it may be possible to limit the number of databases to the minimum set that would have identified all the original included studies. In some reviews, MEDLINE alone will suffice.

Strategies: Search strategies from the original review can be optimized. Investigators should confirm that the search strategies for the retained database would find the included studies from the original review and adjust the search strategies, if necessary, to improve recall. Examples of adjustments would be adding a key subject heading that was omitted from the original search or adding a newly introduced subject heading.[1] After maximizing recall, the searcher should endeavour to optimize precision, removing terms with a low yield of relevant records. Analytic tools such as GoPubMed and PubMed PubReMiner can provide useful analytics for optimizing both recall and precision.[2]

Language: Whilst the original search may include multiple databases of many journals in languages other than English, again examination of the yield of additional studies from other languages can be examined in the original review. In a number of topic areas trials are published only in English language journals, so that a process of searching for non-English language trials may not increase sensitivity of the search. However, this is not currently Cochrane searching policy.

Text word terms: If text word terms are included in the search in the interests of identifying solely not-yet-indexed material, restrict those terms to un-indexed records only, for example, in PubMed the string (pubstatusaheadofprint or publisher[sb] or pubmednotmedline[sb]) can be used.[3, 4]

Using a PubMed-only bespoke search strategy for an update:

If the original included studies are all indexed in MEDLINE and the original search process was robust (for example, involved two or more databases and at least one non-database method for identifying relevant studies) a PubMed-only update can be considered. This would consist of two PubMed searches. The first would be a narrow Boolean search of the main MeSH for the population combined with the main MeSH for the intervention. The second would be a search using the Related Articles feature with the PubMed IDs (PMIDs) of the three newest and three largest included studies as the seed articles.[5, 6]

Can searches for updates be limited update by date?

Where the date of the original searches is known, and the original searches were well-designed and well-conducted, the update search should probably be limited to material added to the relevant databases since the original searches were conducted.[7] At a meeting with US National Library of Medicine (NLM) staff to discuss updating searches using PubMed, they advocated use of the Create Date [CRDT] field, i.e. the date the citation record was first created (McGhee M and Zipser J, oral communication, 20th June 2014).

Where the date of the original search is unknown, one approach is to update the search to include the six months prior to the record creation of the newest included study. In all cases, however, care should be taken to ensure identification of (a) retracted studies (b) errata and corrected records.[8]

Expanding searches to identify retracted studies or errata/corrections

In all cases, the bibliographic record for included studies from the review being updated should be checked to identify retraction and errata.[8]

Additional update searches for trial registers and grey literature

In line with current Cochrane search guidance, as well as searching databases that contributed to the original review, update searches should query trial registers, typically ClinicalTrials.gov and the WHO portal.[8] As well as

identifying studies with results, investigators should identify completed studies without posted results and in progress studies, which can be listed as "studies awaiting assessment".

If, in the original review, no studies were identified solely through grey literature searches, or they are small or appear only as early studies, it may not be productive to update the grey literature search.

1. Sampson M, McGowan J. Inquisitio validus Index Medicus: A simple method of validating MEDLINE systematic review searches. Res Synth Methods 2011;2(2):103-9.

2. Lefebvre C, Glanville J, Wieland LS, et al. Methodological developments in searching for studies for systematic reviews: past, present and future? Syst Rev 2013;2:78.

3. Duffy S, Misso K, de Kock, et al. Supplementary searches of PubMed to improve currency of MEDLINE (Ovid) searches for systematic reviews: time taken for record to move to MEDLINE. Paper presented at 15th EAHIL Conference; 6-11 Jun 2016; Seville, Spain. 2016: Oral E7. http://www.bvsspa.es/eahil2016/wp-content/uploads/2016/05/E7.pdf.

4. Thompson JC, Quigley JM, Halfpenny NJ, et al. Importance and methods of searching for E-publications ahead of print in systematic reviews. Evid Based Med 2016;21(2):55-9.

5. Sampson M, de Bruijn B, Urquhart C, et al. Complementary approaches to searching MEDLINE may be sufficient for updating systematic reviews. J Clin Epidemiol 2016; pii: S0895-4356(16)30016-6. doi: 10.1016/j.jclinepi.2016.03.004.

6. Sampson M, Shojania KG, McGowan J, et al. Surveillance search techniques identified the need to update systematic review. J Clin Epidemiol 2008; 61: 755-62. PubMed doi:10.1016/j.jclinepi.2007.10.003.

7. Bergerhoff K, Ebrahim S, Paletta G. Do we need to consider in process citations for search strategies?. In: Bridging the gaps. Abstracts of the 12th Cochrane Colloquium; 2004 2-6 Oct; Ottawa, Canada. 2004. http://abstracts.cochrane.org/2004-ottawa/do-we-need-consider-process-citations-search-strategies

8. Lefebvre C, Manheimer E, Glanville J. Chapter 6: Searching for studies. In: Higgins JPT, Green S (editors) Cochrane Handbook for Systematic Reviews of Interventions. Version 5.1.0 [updated March 2011]. The Cochrane Collaboration, 2011. Available from www.handbook.cochrane.org.

APPENDIX 3. PUGS Checklist for updating a systematic review: deciding when and how

This is PUGS-1, the first version of the checklist. We will modify this checklist in the light of experiences by a variety of users. Any feedback please contact the corresponding author.

	Criteria	Question	Yes or no	Comment
Whether to update	Step 1. Assess the currency	1. Is it as relevant question?	Yes-go to 2<br>No-no update	Medical progress means some questions become irrelevant
		2. Intervention still in use?	Yes-go to 3<br>No-no update	Some interventions may not be in use in one country, but widely used in another (including the private sector) so consider this.
		3. Research area active with debates in the literature and possibly new evidence emerging?	Yes-go to 5<br>No-go to 4	Areas with equipoise and new trials are a priority to update.
		4. Has the review been used?	Yes-go to 5<br>No-probably not worth updating	If access is considerable, make update a priority. If not well accessed, used, or cited, compared to those in a similar topic, then it may be that this is not an area of equipoise or debate.
		5. Does the review use valid methods and is well conducted?	Yes-go to 6<br>No-start with a new protocol	Assessment of the methods should take into account methods available when the review was conducted
	Step 2. Identify new methods, new studies and other information	6. Are there new studies published or completed?	Yes-go to 9<br>No-go to 7	It helps the reader to know that there are no new studies meeting the inclusion criteria.
		7. Has new information or data from existing included studies come to light that is useful?	Yes-UPDATE & go to 11<br>No-go to 8	More information on methods, risk of bias or results may improve the review.
	Step 3. Assess impact of updating the review	8. Will application of new synthesis methods substantially improve the quality and clarity of the review?	Yes-UPDATE & go to 11<br>No-no update	GRADE has substantive effects on improving review quality
		9. Will the data in the new studies change the findings of the review, or substantially inform the review?	Yes-UPDATE & go to 11<br>No-go to 10	Use "eyeball" appraisal or formal methods. New studies inform the main desired outcome, but could also inform by being in a different setting, age group, or include new adverse effects
		10. Will the absence of the new studies and the older search date lead to questions about the credibility of the review?	Yes-UPDATE and go to 11<br>No-no update	A large new study may not alter a review's bottom line but damage its credibility if not included; whereas a large review and with a few small recent studies may not.
Preparing to update	Refresh protocol	11. Given advances in the field, do the existing objectives, review PICO and methods need modifying?	Yes-adjust & go to 12<br>No-go to 12	Sometimes new interventions may need to be included. Note any broadening of inclusion criteria will need a new search strategy
	Appraise author team	12. Are we/the author team willing, capable and has the time to complete the update?	Yes-go to 13<br>No-find new team & go to 13	Who makes this judgment varies with commissioning agency; new authors or a new team may be required.
	Competing interests	13. Are there any competing interests, such as review authors also investigators of included trials?	Yes-deal with, then go to 14<br>No- go to 14	Competing interests, including academic, can substantially influence reviews and should be managed carefully. For current and new authors, take into account developments in managing competing interests
	Refine search	14. Does the search need to be altered in the light of change in PICO, or can be improved using the yield from the search of the current published version?	Yes-modify & to go to 15<br>No-go to 15	—
	—	15. Author team start update	—	—

Date: 13th July 2016
